# Heterologous Infection of Pregnant Mice Induces Low Birth Weight and Modifies Offspring Susceptibility to Malaria

**DOI:** 10.1371/journal.pone.0160120

**Published:** 2016-07-28

**Authors:** Ankur Sharma, Solomon Conteh, Jean Langhorne, Patrick E. Duffy

**Affiliations:** 1 Laboratory of Malaria Immunology and Vaccinology, National Institute of Allergy and Infectious Diseases, National Institutes of Health, Rockville, MD, United States of America; 2 Francis Crick Institute, Mill Hill Laboratory, London, United Kingdom; Instituto de Ciências Biomédicas / Universidade de São Paulo - USP, BRAZIL

## Abstract

Pregnancy malaria (PM) is associated with poor pregnancy outcomes, and can arise due to relapse, recrudescence or a re-infection with heterologous parasites. We have used the *Plasmodium chabaudi* model of pregnancy malaria in C57BL/6 mice to examine recrudescence and heterologous infection using *CB* and *AS* parasite strains. After an initial course of patent parasitemia and first recrudescence, *CB* but not *AS* parasites were observed to recrudesce again in most animals that became pregnant. Pregnancy exacerbated heterologous *CB* infection of *AS-*experienced mice, leading to mortality and impaired post-natal growth of pups. Parasites were detected in placental blood without evidence of sequestration, unlike *P*. *falciparum* but similar to other malaria species that infect pregnant women. Inflammatory cytokine levels were elevated in pregnant females during malaria, and associated with intensity of infection and with poor outcomes. Pups born to dams during heterologous infection were more resistant to malaria infections at 6–7 weeks of age, compared to pups born to malaria-experienced but uninfected dams or to malaria-naïve dams. In summary, our mouse model reproduces several features of human PM, including recrudescences, heterologous infections, poor pregnancy outcomes associated with inflammatory cytokines, and modulation of offspring susceptibility to malaria. This model should be further studied to explore mechanisms underlying PM pathogenesis.

## Introduction

50 million women from malaria endemic countries become pregnant each year, and risk malaria-related sequelae such as severe maternal anemia, stillbirth, low birth weight and neonatal death [1, 2]. *Plasmodium falciparum* malaria during pregnancy is characterized by placental sequestration of infected erythrocytes, a feature not observed with other parasite species such as *Plasmodium vivax*. Sequestration of *P*. *falciparum* parasites is mediated by binding to chondroitin sulfate A (CSA) in the placenta [3], which distinguishes these parasites from those obtained from non-pregnant individuals. The binding of infected erythrocytes to CSA is linked to the expression of VAR2CSA, a distinctly structured member of the *P*. *falciparum* erythrocyte membrane protein 1 (PfEMP1) variant surface antigen family [4, 5]. Susceptibility to both *P*. *falciparum* and *P*. *vivax* malaria is greatest in first pregnancies, and decreases over successive pregnancies. Resistance to *P*. *falciparum* increases as women develop antibodies that block adhesion of CSA-binding parasites, and the mechanism for resistance to *P*. *vivax* is unknown.

Our understanding of pregnancy malaria (PM) has largely come from studies of women in endemic areas, but progress has been made developing animal models that capture features of the human experience. In BALB/c mice, *P*. *berghei* causes severe placental pathology with decreased fetal viability, intra-uterine growth retardation and gross post-natal growth impairment [6], and shares features of human malaria such as recrudescence during pregnancy and adhesion to placenta [7]. Because *P*. *berghei* infection in rodents is uniformly lethal [8, 9], the animals require prompt treatment, which limits comparisons to malaria-experienced women who frequently have asymptomatic infections that remain untreated and for *P*. *falciparum* which can progress to chronic placental malaria.

*Plasmodium chabaudi chabaudi (Pcc)*, another rodent parasite studied in pregnant mice, offers several parallels to *P*. *falciparum*. Unlike *P*. *berghei* (and *P*. *vivax*) which preferentially infects reticulocytes, *P*. *chabaudi* infects erythrocytes of all ages [10], can cause chronic and recrudescent infection, and has virulence-associated properties like rosetting and sequestration in deep vascular beds [11–14]. Genetically distinct isolates of *P*. *chabaudi* allow studies of mixed and heterologous infections (HI) [15, 16]. In malaria-naïve pregnant C57BL/6 mice, *P*. *chabaudi* AS (*Pcc-*AS) causes spontaneous abortions, fetal loss and parasite accumulation in the placenta [17]. As with human falciparum infections, pregnancy loss caused by *Pcc-*AS infection is associated with placental and peripheral pro-inflammatory cytokines responses in both A/J and C57BL/6 mice [18, 19].

The aim of the present study was to establish a rodent model of parasite recrudescence and heterologous reinfection in malaria-experienced dams. We show that the more virulent *Plasmodium chabaudi* CB (*Pcc-*CB) infection causes parasite recrudescence accompanied by anemia during pregnancy in C57BL/6 mice. HI of *Pcc-*AS-exposed mice with *Pcc-CB* during pregnancy leads to severe pregnancy outcomes associated with pro-inflammatory cytokines, and pups born to infected dams had impaired growth and altered malarial susceptibility. We did not observe placental sequestration of *Pcc-*CB during either recrudescence or HI.

## Materials and Methods

### Mice

6–8 week old C57BL/6 mice were obtained from Taconic or the National Institutes of Health (NIH). Mice were kept in reverse light conditions with 12-hr dark and 12-hr light cycles. For pregnancy studies, 1 female was mated with 1 male and fed with breeder diet for at least a week before pairing with males. For data consistency, the non-pregnant control animals were also fed the same breeder diet. All female mice were weighed before mating and then daily to confirm successful pregnancy. First observation of vaginal plug was considered as gestation day 0 (GD 0) of pregnancy. Vaginal plugs and weight gain after mating were considered to be true markers for successful pregnancy and weight loss was considered as a marker for abortion. No animals died due to infection or reinfection. No more than 10 animals were treated for malaria according to veterinarian’s recommendations due to signs of distress and parasitemia, and therefore were removed from the study. Animals were sacrificed using a CO_2_ chamber and cervical dislocation. Animal studies were performed following the guidelines provided by the National Institutes of Health (NIH) Animal Care and Use Committee (ACUC) and approved by the NIH ACUC, according to the Institutional Animal Care and Use Committee (IACUC) approved protocol. The National Institutes of Allergy and Infectious Disease (NIAID), Division of Intramural Research (DIR) Animal Care and Use Program, as part of the NIH Intramural Research Program (IRP), complies with all applicable provisions of the Animal Welfare Act and other Federal statutes and regulations relating to animals.

### Parasites and Mosquitoes

Cloned lines of *Plasmodium chabaudi chabaudi* (*Pcc*) strains, *Pcc-*AS and *Pcc-*CB were obtained from the MRC National Institute of Malaria Research, Mill Hill, UK. Parasites were maintained as frozen stocks and passaged in C57BL/6 mice when needed for infection. To obtain *P*. *chabaudi* sporozoites, female mosquitoes (*Anopheles stephensi*) were fed on infected C57BL/6 mice and then maintained for 14–16 days when sporozoites were harvested from salivary glands, as described elsewhere [15].

### Experimental Design

We first defined the typical course of *Pcc-*AS and *Pcc-*CB blood stage infection in non-pregnant BL6 mice housed at our facilities. Mice were injected intravenously with 100,000 infected murine red blood cells (iRBCs) or 2000 *P*. *chabaudi* sporozoites (Spz) of either *Pcc-AS* or *Pcc-CB*. Animals were monitored daily by Giemsa-stained blood smears for up to 90 days to detect and quantify parasitemia.

For parasite recrudescence studies, female mice were infected intravenously with either *Pcc-*AS or *Pcc-*CB iRBCs or Spz, and then mated with males 35 days post infection. Vaginal plugs and daily weights were monitored to confirm pregnancy and to determine gestational age. Blood smears were not made for a week after pairing to minimize stress and increase the chance of successful mating. One week after pairing, daily blood smears were initiated to detect parasite recrudescence in the pregnant animals. Infected non-pregnant controls were also handled in a similar manner.

For studies of re-infection with heterologous parasites, female mice were infected intravenously with 1000 *Pcc-*AS Spz, then mated with males 35 days post-infection. Vaginal plugs and daily weights were monitored to confirm pregnancy and to determine gestational age. One week after pairing, parasitemia was monitored by daily blood smears. At gestational day 10, female mice were infected intravenously with 100,000 *Pcc-*CB iRBCs and daily blood smears were continued till delivery, to determine parasitemia. Blood was also collected at gestational day 18 for complete blood count analysis. Uninfected pregnant and infected non-pregnant controls were also monitored and handled in a similar manner.

### Placenta processing and follow up of offspring

Half of the pregnant mice from each group were sacrificed at gestational day 19 to collect placentas. Half of these placentas were fixed in 4% paraformaldehyde (PFA), embedded in paraffin and stained in hematoxylin and eosin (HE) and with Giemsa for placental histology. The other placentas were ground using a mechanical homogenizer at low settings so as to not disrupt the RBCs. The homogenized mixture was then passed sequentially through a size 70 and size 40 cell strainer to collect blood for thin and thick Giemsa stained blood smears to determine placental parasitemia.

The remaining pregnant mice from each group were allowed to progress to delivery. Litter size, newborn weight and viability, abortions, and maternal mortality were recorded. Because we were interested in long-term outcomes of pups, we did not separate pups from dams for weighing or other manipulations until day 5 post-delivery. A stillbirth was defined as the absence of any viable pups at the time of delivery or euthanasia, and neonatal death was defined as the death of a pup 1 day after birth. When the pups were 7–8 weeks old, they were intravenously infected with 1000 *Pcc-*CB sporozoites and the parasitemia was followed for up to 30 days by daily blood smears.

### Cytokine analysis

Blood was collected periodically throughout the experiment. Plasma was separated from blood and stored at -80°C until assayed for IL-6, IL-10, IL-12, TNF-α, IFN-γ and MCP-1 using the commercially available Milliplex Map Mouse Cytokine/Chemokine kit from EMD Millipore, USA. Cytokines were measured at sequential time-points before and during infection, prior to pregnancy, at gestational day 18 for all pregnant mice, and simultaneously in re-infected non-pregnant control mice. The plate was run on Luminex 100 and the data analyzed by interpolating the standard curve using median fluorescent intensities.

### Statistics

Statistical analyses were performed using GraphPad Prism version 6.0g for Mac. The parasitemia levels (% infected red cells) in different groups of mice are expressed as mean with 95% confidence intervals. *P* values, unless detailed in the figure legends, were calculated using Welch’s unpaired t-test and *P* ≤ 0.05 was considered as statistically significant. To compare hemoglobin and hematocrit levels, as well as cytokine levels, data were analyzed by one-way ANOVA using Bonferroni test to correct for multiple comparisons. To calculate the difference in the weight of pups, a generalized linear model was used which takes into account the correlation due to repeated measures within pups, and the correlation due to multiple pups born to the same dam (PROC MIXED, with a repeat command, and a random term for the mother; SAS 9.3). Differences between groups in the mean values of peak parasitemia were compared using Welch’s unequal variances t-test.

## Results

### Characterization of *Pcc* infection in C57BL/6 mice

*Pcc* infection of laboratory mice has been extensively documented [15, 20]. Briefly, mice infected intravenously with 1 x 10^5^ infected red blood cells (iRBCs) of either *Pcc-*AS or *Pcc-*CB had an average peak parasitemia of 37.8 ± 3.0% (Mean ± SD) or 41.8 ± 4.3% on day 7, respectively, followed by a second modest peak of parasitemia on day 14 (S1A Fig).

Mice infected with 2000 *Pcc-*AS or *Pcc-*CB sporozoites had a first peak parasitemia of 1.1 ± 0.3% or 18.0 ± 1.6%, respectively, on day 11 post-infection (S1B Fig). *Pcc-*CB but not *Pcc-*AS recrudesced as a second episode of patent parasitemia (based on Giemsa-stained blood smears), re-appearing on day 26 post-infection at 1.0 ± 0.4%, and clearing by day 28. No further recrudescences were observed up to day 90 post-infection.

### Parasite recrudescence with *Plasmodium chabaudi* in pregnant mice

**Fig 1:**
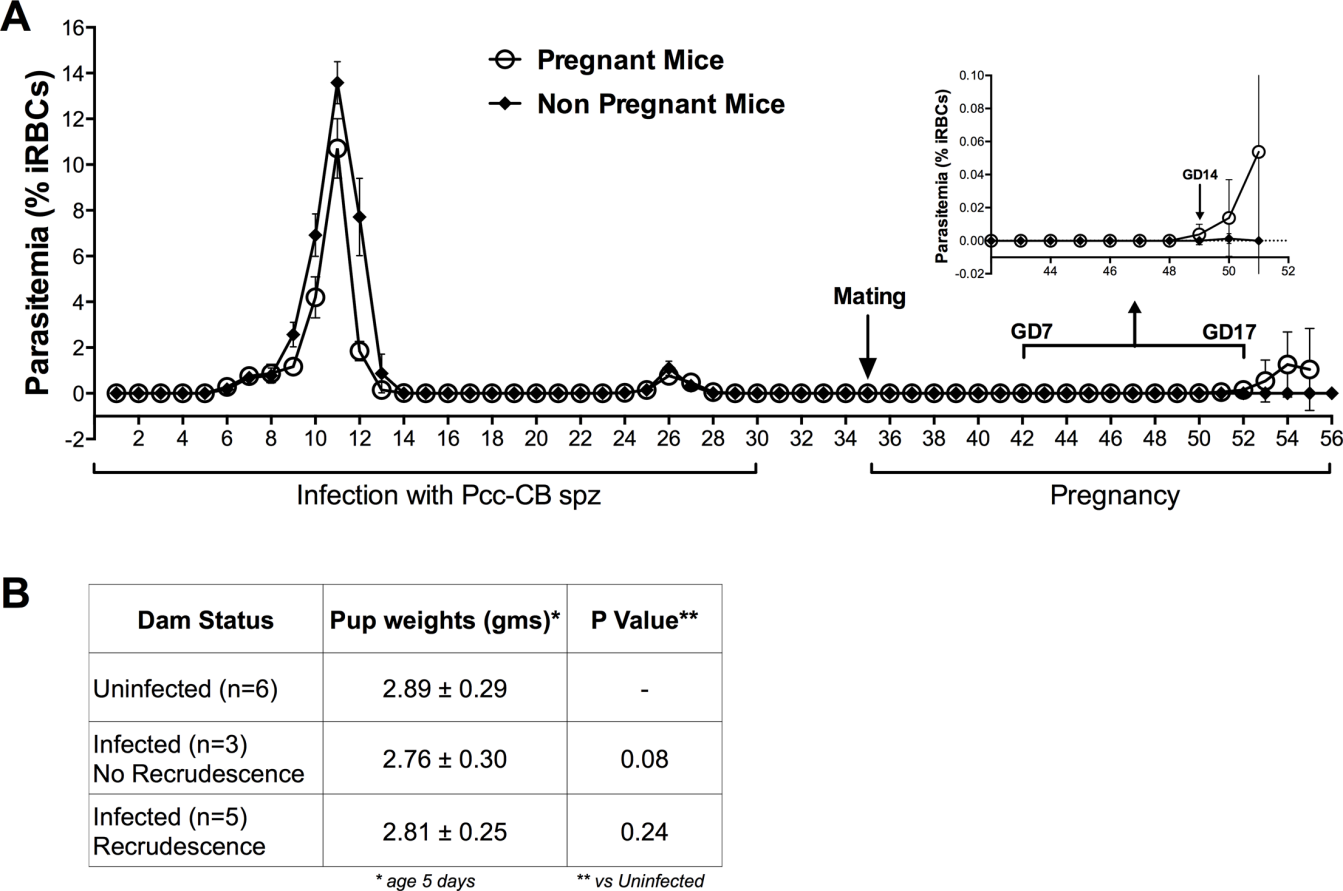
Recrudescence of *P*. *chabaudi* in mice during pregnancy. After a primary infection initiated by inoculation of *Pcc-*CB sporozoites, mice were paired with males at 35 days post-inoculation (“Pregnant Mice”, n = 16) or remained unpaired (“Non Pregnant Mice”, n = 12). Parasitemia was monitored by daily blood smears throughout the study. Data are presented as means with 95% confidence intervals. Experiment was repeated with similar results.

On day 35 after infection with *Pcc-*CB sporozoites, female mice were paired with males and parasitemia was monitored by blood smears starting a week later. 68% of pregnant mice showed parasite recrudescence at or after gestational day (GD) 14 with an average peak parasitemia of 2.6 ± 1.5% (Fig 1A). Parasite recrudescence was never observed before GD14, as shown by the inset in Fig 1A. None of the mice succumbed to the infection or suffered abortions or stillbirths. In addition, pup weights were measured at day 5 of birth, and no significant differences were observed between pups born to infected dams with recrudescence, pups born to infected dams with no recrudescence, and uninfected dams (Fig 1B). The experiment was repeated with similar results. Pregnant mice with parasite recrudescences had 36% lower hemoglobin (*p<0*.*0001*), 43% lower platelet count (*p<0*.*0001*) and 28% lower hematocrit (*p<0*.*001*) at gestational day 18, compared to mice without parasite recrudescence or to un-infected control mice (Table 1). Mice without parasite recrudescence did not differ significantly from un-infected control mice by any of these hematological parameters.

**Table 1:** Hematological profiles of infected and uninfected mice at gestational day 18.

	Uninfected Pregnant Mice (n = 12)	Infected Pregnant Mice (n = 16)	
		No Recrudescence (n = 5)	Recrudescence (n = 11)
WBC (10^9^/L)	3.79 ± 0.4	4.01 ± 0.6, *	4.32 ± 0.9, *
RBC (10^12^/L)	8.48 ± 0.5	8.98 ± 0.5, *	6.62 ± 0.8, **
Hgb (g/dL)	14.4 ± 0.95	14.1 ± 0.9, *	9.1 ± 0.63, ***
HCT (%)	40.9 ± 2.4	41.7 ± 3.5, *	29.4 ± 3.3, **
Platelets (10^9^/L)	1020 ± 103	1125 ± 112, *	581 ± 143, ***

* Not Significant

** p <0.001

*** p<0.0001.

Infected mice (n = 16) received *Pcc-*CB sporozoites and were then paired with males 35 days post-inoculation; age-matched mice that had not been infected (n = 12) were paired with males simultaneously. Hematological parameters, including white blood cell (WBC) count, red blood cell (RBC) count, hemoglobin (Hgb) level, hematocrit (HCT), and platelet count were measured. Data are presented as means ± SD. *P* values were calculated using Welch’s unpaired t-test, comparing subgroups of infected mice to uninfected mice.

Conversely, only 2/20 mice infected with *Pcc-*AS sporozoites (n = 20) experienced parasite recrudescence during pregnancy, and those 2 mice had an average peak parasitemia of 0.015 ± 0.007%. Non-pregnant mice, infected with sporozoites of either strain, showed no patent parasite recrudescence after day 30 of infection (S1A and S1B Fig). Similarly, mice infected with blood stage parasites of either strain did not experience recrudescences during pregnancy (data not shown).

### Re-infection with heterologous *Pcc* causes poor pregnancy outcomes

**Fig 2:**
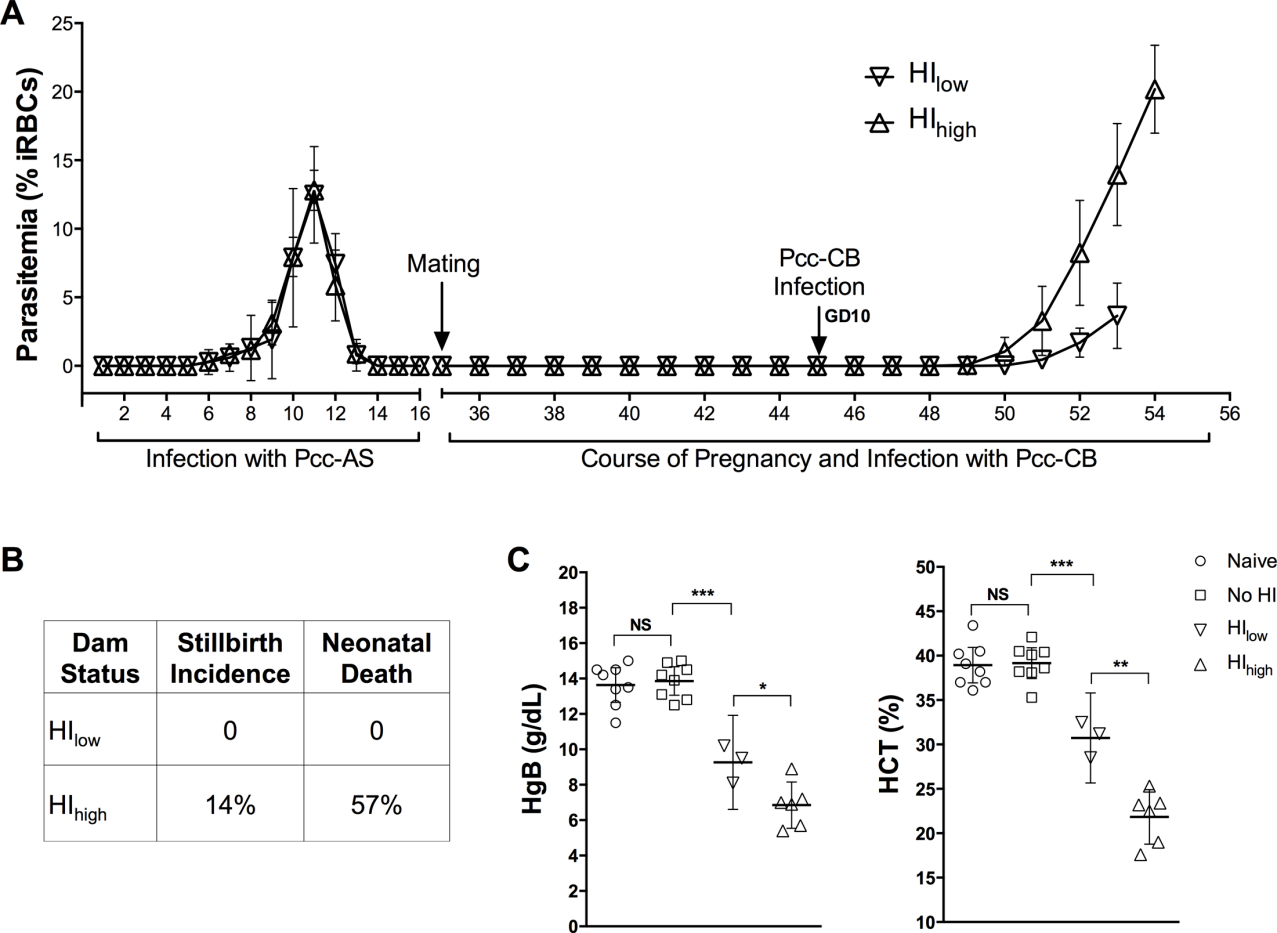
Course of HI in pregnant mice. After a primary infection initiated by inoculation of *Pcc-*AS sporozoites, mice were paired with males 35 days post-inoculation, then randomly assigned to be inoculated (“HI Pregnant”, n = 12) or not inoculated (“No HI Pregnant”, n = 8) with *Pcc-*CB iRBCs at gestational day 10. Control mice (“Naïve Pregnant”, n = 8) were never inoculated with parasites, but were paired with males simultaneously with the infected mice. (A) Parasitemia was monitored during *Pcc-*AS sporozoite-induced infection and *Pcc-*CB iRBC-induced re-infection during pregnancy by daily blood smears. Data are presented as means with 95% confidence intervals. (B) Comparison of Hgb and HCT in different groups of mice as indicated at gestational day 18. Data are presented as means with 95% confidence intervals. Data were analyzed by one-way ANOVA using Bonferroni test to correct for multiple comparisons. *P < 0.01, **P < 0.001, ***P < 0.0001, and NS = not significant. Similar results were obtained in two repeats of this experiment.

Twelve C57BL/6 mice were infected with 2000 *Pcc-*AS sporozoites and paired with males 35 days later. At gestational day 10, mice were inoculated with 10^5^ *Pcc-*CB infected red blood cells (iRBCs) (Fig 2A). 2 out of 12 mice did not get pregnant and one mouse had an abortion at gestational day 16 with 17.3% parasitemia. 3 mice had comparatively low parasite density (HI_low_) with average parasitemia of 3.65 ± 0.95% at gestational day 18 while the remaining 6 mice experienced high parasite density (HI_high_) with average parasitemia of 17.70 ± 4.8% (Fig 2A). High parasite density was also associated with stillbirth (14%) or neonatal death (57%), whereas the HI_low_ group had no incidence of stillbirth or neonatal death (Fig 2B). Both groups of re-infected mice (HI_low_ and HI_high_) had significantly lower hemoglobin and lower hematocrit at gestational day 18 compared to mice that were not re-infected during pregnancy or to naïve pregnant mice (Fig 2C). Further, HI_high_ mice had significantly lower hemoglobin (6.85 g/dL (*p < 0*.*01*)) and hematocrit (21.8% (*p < 0*.*001*)), compared to HI_low_ mice (9.26 g/dL and 30.7%, respectively) at gestational day 18 (Fig 2C).

**Fig 3:**
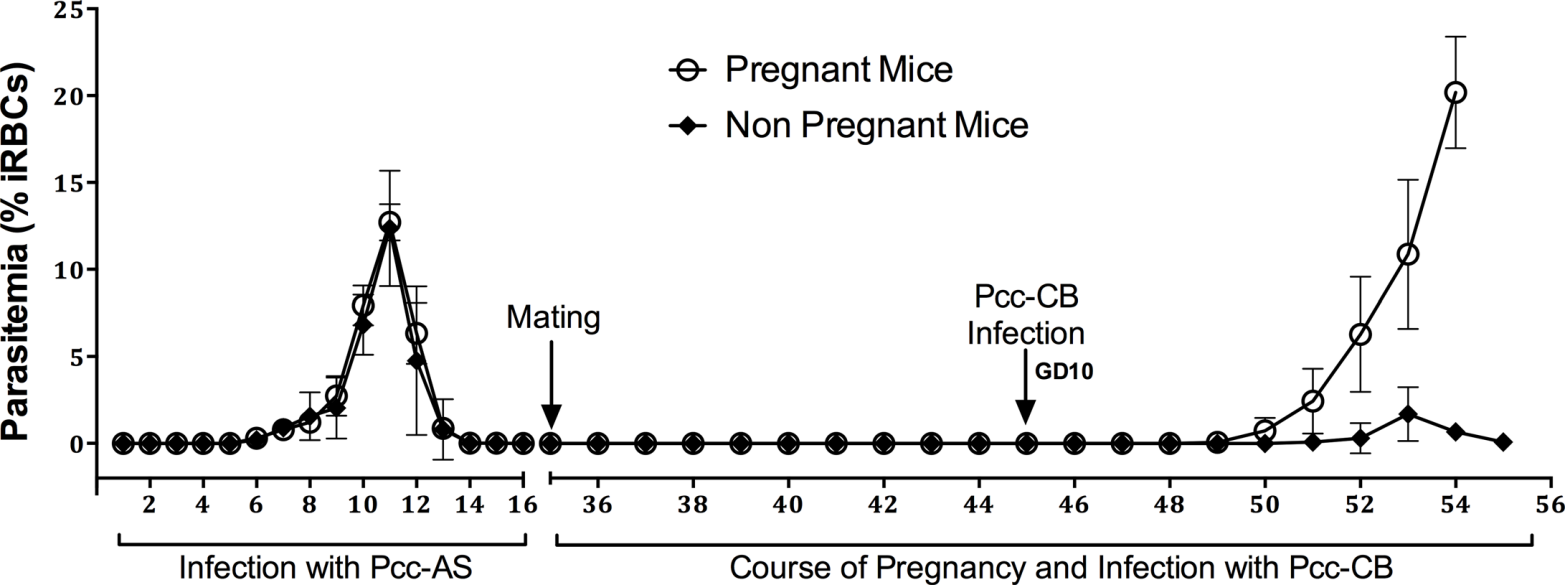
Course of parasitemia in pregnant versus non-pregnant mice re-infected with a heterologous strain of *P*. *chabaudi*. Mice were inoculated intravenously with *Pcc-*AS sporozoites, randomly assigned to be paired or not with males 36 days post-inoculation, and then injected with *Pcc-*CB iRBCs 46 days post-inoculation. Parasitemia was monitored by daily blood smears throughout the study. Data are presented as means with 95% confidence intervals. Similar results were obtained in two repeats of this experiment.

Previously published studies with different rodent parasites have shown that pregnancy increases susceptibility to parasitemia [9, 17, 21]. Mice were infected with *Pcc-*AS Spz, then randomly assigned to be paired or not paired with males (average first peak parasitemia of 12.4 ± 2.1% and 12.7 ± 1.5% in the two groups (Fig 3)). One group was paired with males 35 days post-infection, and both groups received *Pcc-*CB iRBC at gestational day 10. Average peak parasitemia in pregnant mice at gestational day 18 was 14.8 ± 4.8%, versus 1.7 ± 1.0% measured contemporaneously in the unmated mice (Fig 3; *p < 0*.*05*) demonstrating that pregnancy increases susceptibility to *Pcc* re-infections. Control mice (n = 8) infected with *Pcc-*AS sporozoites before pregnancy but receiving no further parasite inoculations did not develop any parasitemia during pregnancy and gave birth to normal pups.

### Peripheral cytokine levels in *Pcc* infected pregnant mice

**Fig 4:**
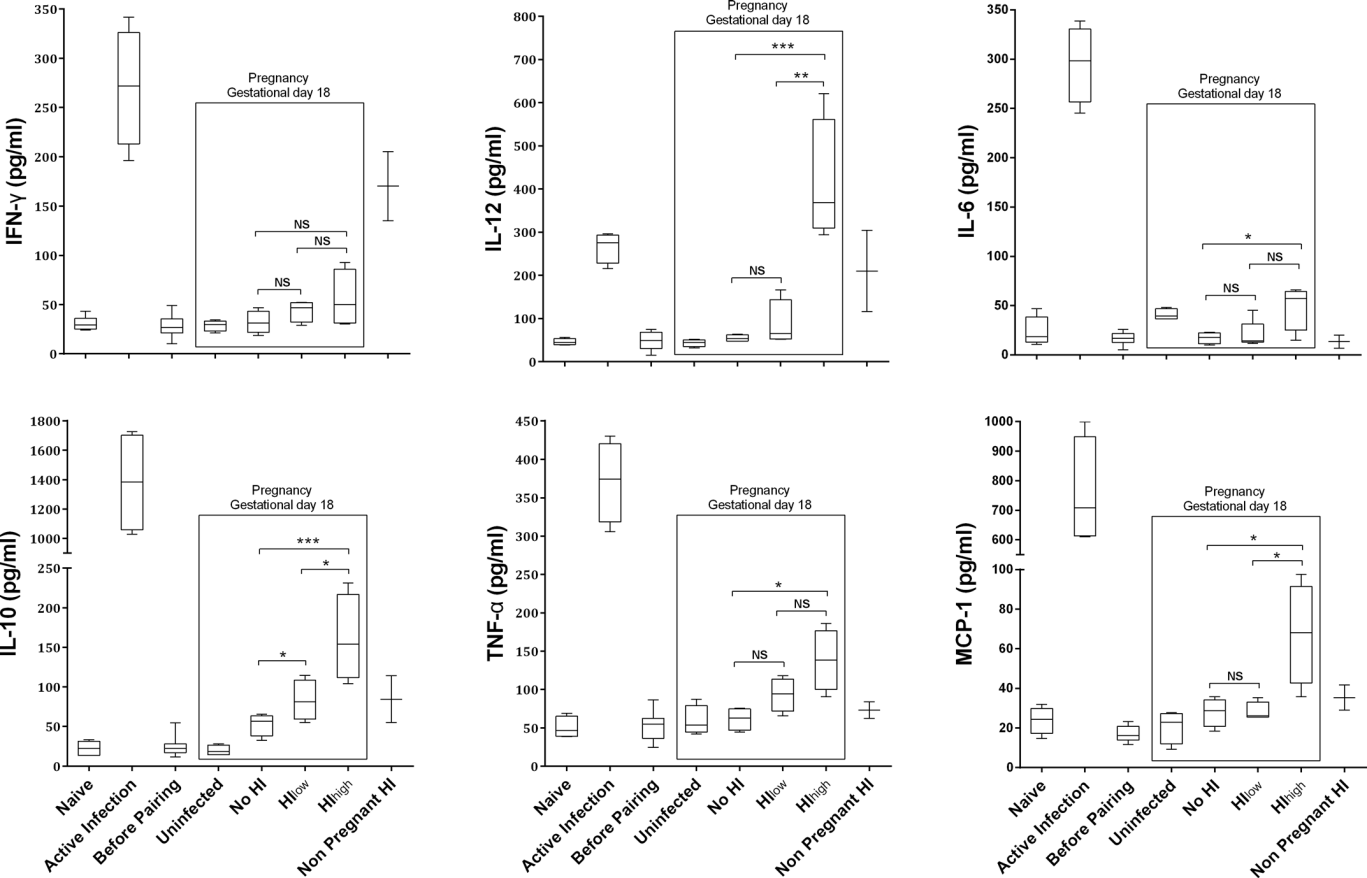
Peripheral cytokine responses in *P*. *chabaudi* re-infected or never-infected pregnant mice, and in non-pregnant re-infected mice. Cytokines were measured at sequential time-points before (“Naïve”) and during infection (“Active Infection”), prior to pregnancy (“Before Pairing”), and then at gestational day 18 for all pregnant mouse groups (“Uninfected”, “HI_low_”, “HI_high_” and “No HI”) and simultaneously in re-infected non-pregnant control mice (Non Pregnant HI). (A-F) Cytokines IFN-γ, IL-12, IL-6, IL-10, TNF-α and MCP-1 were measured using Milliplex Map Mouse cytokine kit. Data were analyzed by one-way ANOVA using Bonferroni test to correct for multiple comparisons, and are presented as box and whiskers plots showing min and max values. *P < 0.05, **P < 0.01, ***P < 0.001 and NS = not significant.

Following *Pcc-*AS sporozoite inoculation in non-pregnant animals, peripheral blood levels of IL-12, IL-10, IL-6, TNF-α, IFN-γ and MCP-1 were measured at sequential time-points before (“Naïve”) and during infection (“Active Infection”), prior to pregnancy (“Before Pairing”), at gestational day 18 for all pregnant mouse groups (“Uninfected”, “HI_low_”, “HI_high_” and “No HI”) and simultaneously in re-infected non-pregnant control mice (Non Pregnant HI) (Fig 4). All cytokine levels were significantly higher at active infection (or at peak parasitemia) compared to levels in uninfected control mice (Fig 4), as has been described previously [22]. By 30 days post-infection, all cytokines returned to their baseline levels.

During HI of pregnant mice, some but not all cytokines again increased, with a more pronounced response in HI_high_ animals. The cytokine levels in the control group of pregnant mice (infected before but not during pregnancy) were comparable to cytokine levels in pregnant mice that had never been infected (Fig 4). At gestation day 18, TNF-α and MCP-1 levels were elevated in HI_high_ pregnant mice (2.2-fold and 2.4-fold increases, respectively; both *p < 0*.*05*), but were not elevated in HI_low_ pregnant mice, compared to the “No HI” group of pregnant mice (Fig 4). Increases in IL-10 and IL-12 levels were even greater (3.1-fold and 7.5-fold increase, respectively; both *p < 0*.*005*) in HI_high_ pregnant mice compared to “No HI” pregnant mice. Interestingly, the non-pregnant animals but not the pregnant animals (HI_low_ and HI_high_) experienced significant increase in IFN-γ during HI.

### Placental changes during *Pcc* infections

**Fig 5:**
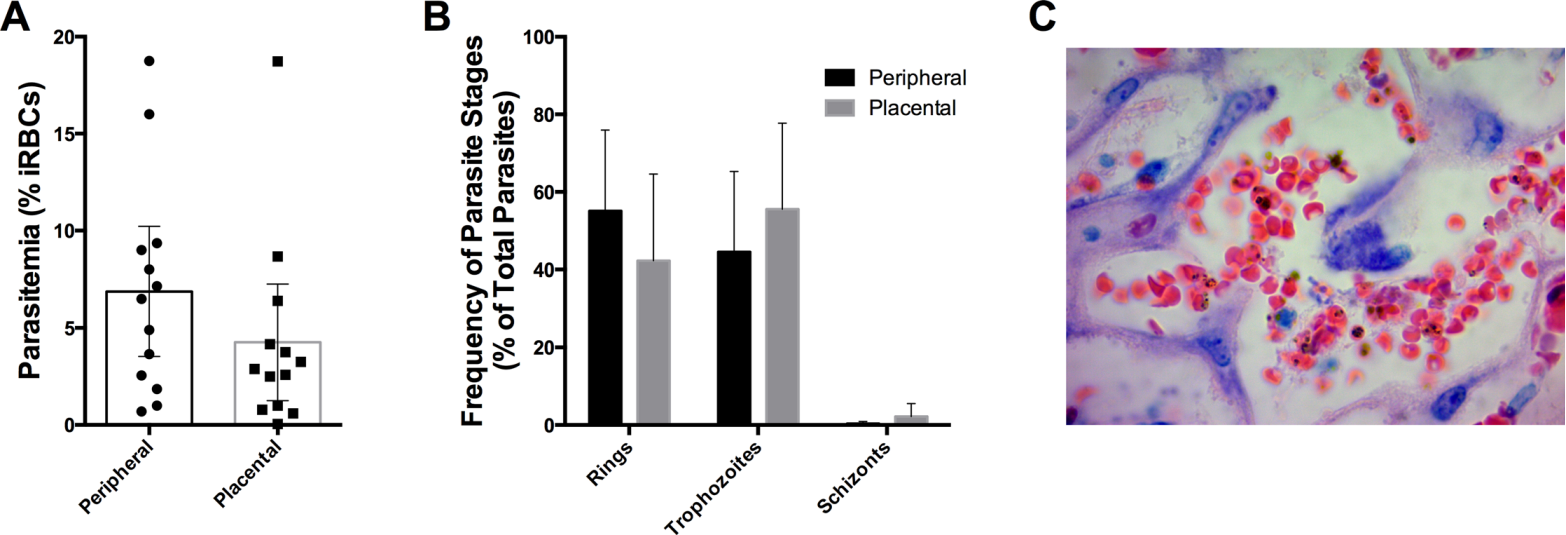
Peripheral and placental parasitemia during *P*. *chabaudi* re-infection at gestational day 18. (A) Parasite burden in the peripheral and placental blood of mice 8 days after re-infection with heterologous *Pcc-*CB iRBCs. (B) Frequency of different parasite stages in the peripheral and placental blood of mice 8 days after re-infection with heterologous *Pcc-*CB iRBCs. (C) Giemsa-stained placental tissue section from a mouse 8 days after re-infection with heterologous *Pcc-*CB iRBCs. Data are presented as means with 95% confidence intervals.

Mice were housed in a room with reverse 12 hour light-dark cycle in order to align the highly synchronous *Plasmodium chabaudi* life cycle with blood sampling for the determination of sequestration. We assessed evidence for the accumulation or sequestration of iRBCs in placenta, by comparing the peripheral and placental parasitemias scored on Giemsa-stained blood smears (Fig 5A and 5B) and by examining placental sections (Fig 5C), for evidence that parasites in the placenta appeared at a higher density and with an increased proportion of mature stage parasites. In the pregnant mice that experienced recrudescence of *Pcc-*CB, there was no significant difference in placental and peripheral parasite levels (data not shown). We also compared the peripheral and placental parasite densities in pregnant mice during HI with *Pcc-*CB, and did not observe any significant difference (Fig 5A). Most of the parasites were ring and trophozoite forms, and mature schizont forms were rare in both peripheral and placental blood (Fig 5B). In examinations of placental tissue sections, we detected iRBC (Fig 5C) but did not observe any abnormal placental tissue architecture or the accumulation of inflammatory cells in the infected pregnant mice. Of note, we were not able to collect placentas from mice that had an abortion or from the mice that gave birth to dead pups.

To confirm these findings and ensure sampling during the time of *Pcc* sequestration, we repeated the experiment using a larger number of mice (n = 30). We collected peripheral blood smears and placentas (n = 5) every hour at 6 different time points from 11 am to 4 pm. We compared Giemsa-stained peripheral and placental blood smears and placental sections from all these time points, and consistently observed similar parasite densities in peripheral and placental blood, and no evidence of sequestration (data not shown).

### Heterologous infection during Pregnancy causes Persistent Low Weight in Pups

**Fig 6:**
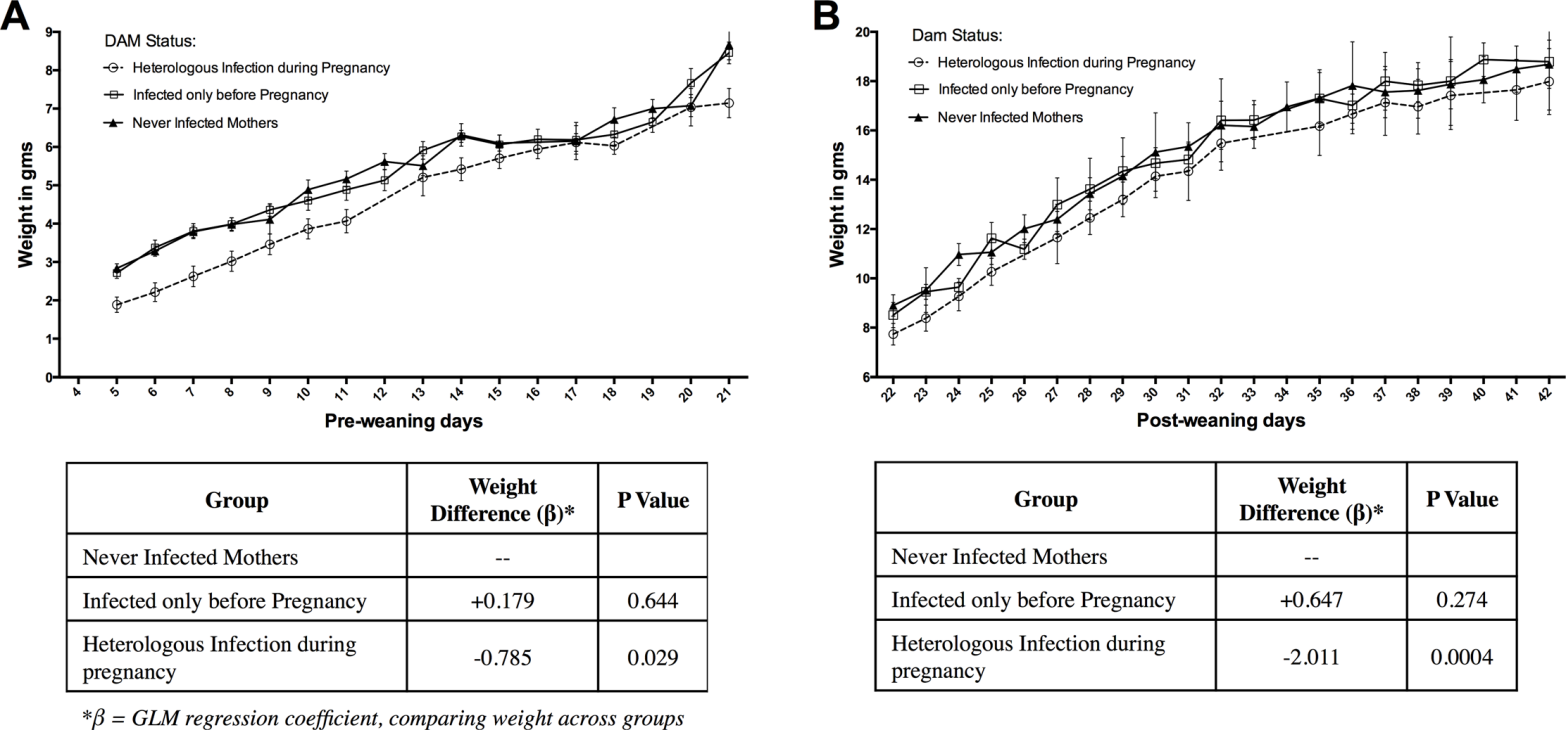
Weight gain in pups born to dams that received *Pcc-*AS before and *Pcc-*CB during pregnancy, dams that received *Pcc-*AS before pregnancy and no other infection during pregnancy, or dams that were never infected. Weights of pups were documented for up to 42 days after birth. (A) Weight in grams throughout the pre-weaning period. (B) Weight in grams throughout the post-weaning period. Data are presented as means with 95% confidence intervals. Differences in weight were analyzed by creating a generalized linear model while taking into account the correlation due to repeated measures within pups and due to multiple pups born to the same dam. β = GLM regression coefficient comparing weight across groups.

Among ten C57BL/6 mice that experienced HI, one mouse had an abortion at gestational day 16. Five of these mice experienced high parasite density, and did not produce viable pups. Four mice (3 low parasite density, 1 high parasite density) had normal delivery and gave birth to a total of 27 viable pups. Pup weights were documented from day 5 up to 42 days after birth and compared to those of pups born to dams without HI (n = 29) or to never-infected dams (n = 34) (Fig 6A and 6B). Litter sizes did not significantly differ between groups (means of 6.8, 9.7, and 8.5 pups, respectively).

A generalized linear model was used to estimate the difference in weight between groups. Two models were specified, one for the pre-weaning period: days 5 through 21, and one for the post-weaning period: days 22 through 42. After controlling for within-pup and within-dam correlation, the “heterologous infection” group was significantly smaller than the “never-infected” group on days 5–21 (β = -0.79 g; p = 0.029) (Fig 6A), and on days 22–49 (β = -2.01 g; p = 0.0004) (Fig 6B).

### Heterologous infection during Pregnancy alters Pup Susceptibility to Malaria

Next we examined the susceptibility of the pups to *Pcc* infection, and compared those born to dams during HI with those born to dams that were never infected or were infected only before their pregnancy. We challenged 7–8 week old pups with 1000 *Pcc-*CB sporozoites intravenously and monitored the parasitemia over a period of 32 days by scoring parasitemia with Giemsa-stained blood smears every 24 h. Differences in parasitemia between groups were evaluated with linear mixed-effects models, allowing for quadratic time trends and mouse-specific random effects, over the course of infection (i.e. days 6 through 20). Differences in the mean values of peak parasitemia between groups were compared using Welch’s unequal variances *t-*tests.

**Fig 7:**
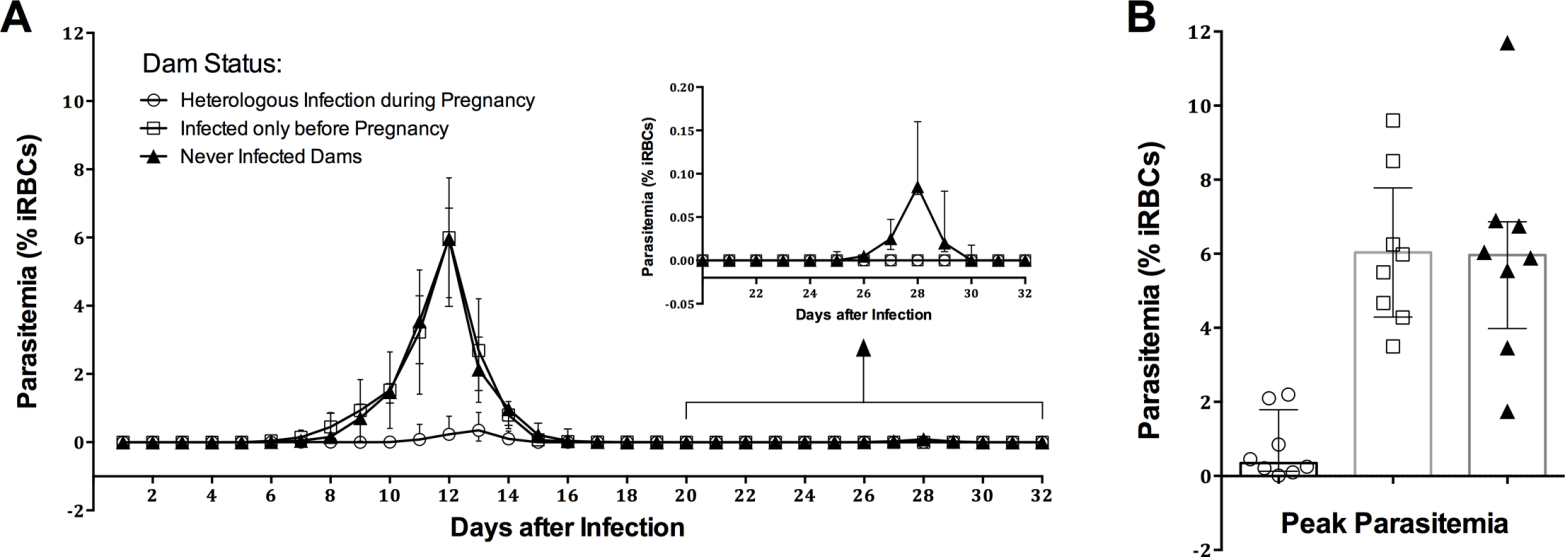
Course of *P*. *chabaudi* infection differs between pups born to infected versus uninfected dams. (A) Pups, born to dams with different infection histories (indicated by icons in the figure), were infected with *Pcc-*CB sporozoites, and peripheral parasitemia followed for up to 32 days; (A, inset) parasite burden between days 22 and 32. (B) Peak parasite burden in pups born to dams that were given HI versus pups born to mothers that were not given HI or were never infected. Data are presented as means with 95% confidence intervals. Differences between groups in the mean values of peak parasitemia were compared using Welch’s unequal variances t-test.

We observed that pups that had been born to dams during HI had significantly lower parasite burdens when infected at 7–8 weeks of age (*p < 0*.*001*), compared to pups born to malaria-naïve dams (Fig 7A). At peak parasitemia, the difference of means between groups was 5.19 (95% CI: 2.76, 7.62; *p = 0*.*001*) (Fig 7B). On the contrary, we did not find any significant difference in parasite burdens between the pups born to malaria-naïve dams and those born to malaria-experienced dams that were not re-infected during pregnancy (p = 0.903) (Fig 7A); at peak parasitemia, the difference of the means between these groups was 0.03 (95% CI: -2.73, 2.67; *p = 0*.*981*) (Fig 7B). Of note, between days 25 and 30, all 8 pups born to malaria-naïve dams experienced recrudescent infections; no pups in the other groups experienced recrudescences (Fig 7A, inset). During recrudescent infections, the mean value of peak parasitemia in the control group was 0.11 (95% CI: 0.06, 0.15).

## Discussion

In this study, recrudescences or re-infections with *P*. *chabaudi* during pregnancy are accompanied by elevated peripheral levels of inflammatory cytokines; the greatest elevations are seen in re-infected mice with the highest parasite densities, and only this group (HI_high_) suffered poor pregnancy outcomes. Infected RBCs are detected in placental blood, but we found no evidence for placental sequestration; placental sequestration is a typical feature of *P*. *falciparum* but not other species such as *P*. *vivax*. HI of dams results in low weight but increased malaria resistance of their pups during early life, in patterns similar to what has been observed in human studies.

We used *P*. *chabaudi* sporozoites rather than iRBCs to initiate all first infections in our experimental model, as this mimics natural infections and also allowed more detectable parasite recrudescences [15]. Here, we described the patterns of recrudescences during pregnancies that followed *Pcc-*CB Spz infections. Sporozoites however did not induce patent parasitemias in previously exposed animals, therefore iRBC inoculations were employed to produce HI. To our knowledge, this is the first demonstration of a patent re-infection during pregnancy of malaria-experienced dams, making this model more relevant to the human experience, albeit re-infection required iRBC rather than sporozoite inoculation which would have been preferred as the natural route. Similar to humans, pregnant animals were more susceptible to recrudescences and re-infections than non-pregnant animals.

Following sporozoite infection with *Pcc-*CB, most mice experienced a non-lethal but patent parasite recrudescence during subsequent pregnancy. This is similar to early studies showing *P*. *berghei*-infected mice experience parasite recrudescence during pregnancy, but in those experiments most recrudescent infections were lethal [23, 24]. *Pcc-*CB recrudescences uniformly occurred after gestational day 14 (third trimester). In humans, susceptibility to malaria increases after about week 10–12 of pregnancy (second trimester), though parasitemia can be seen either early [25, 26] or late [27] during pregnancies. In a previous study of pregnant women living in a holoendemic area of western Kenya, malaria prevalence increased rapidly at 13–16 weeks gestation (early second trimester) [28]. We also observed significant anemia in mice with parasite recrudescence during pregnancy (Table 1), similar to humans in whom anemia and pregnancy malaria are closely associated [29, 30].

In endemic areas, women have multiple malaria episodes in their lifetime, and experience recrudescences and re-infections during pregnancy. To model these features, we infected mice with the more virulent *Pcc-*CB strain to induce parasitemia before pregnancy, and then recrudescence during gestation; or, we infected mice with the less virulent *Pcc-*AS strain before pregnancy, and then re-infected by inoculation of blood stage *Pcc-*CB parasites during pregnancy. Notably, *Pcc-*CB sporozoite inoculations did not induce new parasitemias in pregnant mice that had been exposed to *Pcc-*AS parasites before pregnancy (data not shown). During HI with *Pcc-*CB iRBC, pregnant mice developed more severe infections than non-pregnant mice: mice exposed to *Pcc-*AS and then infected at gestational day 10 with *Pcc-*CB, often experienced high parasite density at term leading to severe pregnancy outcomes such as pup mortality.

Pregnancy has been described as an immunomodulated state during which both anti-inflammatory cytokines (IL-4 IL-5 and IL-13) and pro-inflammatory cytokines (TNF-α, IFN-γ, and IL-6) are elevated [31–34]. These changes are believed to play a critical role for successful pregnancy, and cytokine balance remains paramount during pregnancy, as excessive inflammatory cytokines may jeopardize the pregnancy [35–37]. This regulation is mediated in part by the anti-inflammatory cytokine, IL-10. Based on gene knock-out studies in mice, IL-10 is not required for fetal development, but plays a vital role in placental growth and remodeling through inhibition of pro-inflammatory cytokines and certain chemokines [35, 36, 38].

In humans, TNF-α is associated with adverse pregnancy outcomes such as pre-term delivery, maternal anemia and low birth weight [39, 40]; in murine studies, TNF-α has been associated with fetal loss [21, 41]. Consistent with these observations, we found HI_high_ mice displayed increased plasma levels of TNF-α (Fig 4), and also experienced increased stillbirth incidence and neonatal death (Fig 2B), as well as more severe maternal anemia (Fig 2C). Plasma levels of MCP-1 and IL-10 also increased in infected pregnant mice, especially HI_high_ mice. Although peripheral cytokine levels do not necessarily reflect placental levels, the similarities in cytokine profiles to those of human PM suggest that this *Pcc* pregnancy model may be useful for dissecting inflammation-induced pathology.

Abnormalities in the placental architecture such as disorganization of labyrinthine zone, distension and disarrangement of perivascular space, massive chronic intervillositis by monocytes/macrophages and accumulation of malarial parasites in the placenta have been associated with severe pregnancy outcomes in both rodents and humans [6, 17, 42–44]. Mature forms of *P*. *chabaudi* accumulate in the spleen and extensively sequester in the lungs and liver [14], and have been reported to accumulate in the placenta [19]. We examined placental sections of mice during recrudescence or HI, and identified *Pcc* infected RBCs. However, unlike *P*. *falciparum*, which can sequester abundantly in the human placenta, we did not find evidence of *Pcc* parasites to sequester in mouse placenta. Parasite sequestration in organs is facilitated by binding of parasite proteins expressed on iRBCs to corresponding receptors on endothelial cells [45]. The human parasite, *P*. *vivax*, as well as rodent parasites including *Pcc* lack the *var* family of genes [14, 46], especially the gene *VAR2CSA* that mediates parasite binding to chondroitin sulfate A in the human placenta [3, 47]. Of note, the *vir* multigene family of *P*. *vivax*, which has been suggested to be a possible mediator of adhesion, shares several orthologues called *cir* in *Pcc* [46, 48, 49]. Therefore, it is possible that *Pcc* may utilize similar adhesion molecules as *P*. *vivax*, and this may account for their similar adhesion patterns.

Malaria during pregnancy has adverse effects on infants, such as intra-uterine growth retardation, stillbirth, preterm delivery and low birth weight [1, 50, 51]. One proposed mechanism for these effects is placental insufficiency due to restriction of blood flow in the placental vessels [6, 51]. In malaria endemic areas, low birth weight and fetal growth restriction have been associated with inflammatory infiltrates and massive chronic intervillositis [52–55]. In our study, we did not observe any placental abnormalities or monocyte infiltrates in the placenta of mice. Nevertheless, pups born of mice during HI suffered growth deficits, compared to pups born of uninfected naïve mothers. Of note, we first measured pup weight on day 5 post-delivery in order to ensure pup survival, and therefore the low weight in the pups of infected dams may have been due to low birth weight as well as access to feeding of ill dams. These findings might be similar to *P*. *vivax* malaria where infections and placental abnormalities are less severe than those of *P*. *falciparum*, but are still found to be associated with maternal anemia, intra-uterine growth restriction and low birth weight [56–58]. Factors other than placental sequestration, such as inflammatory cytokines and maternal anemia, have also been found to be associated with poor pregnancy outcomes [34, 39, 40, 59]. In humans, low birth weights are associated with severe anemia and increased TNF-α as a result of malaria infection in pregnancy [34, 60]. Similar observations have been made in rodent models [18, 21]. In our study, we found low hemoglobin and hematocrit levels as well as elevated inflammatory cytokines in pregnant mice during HI, thus these factors may be contributing to the low weight in pups.

Passive transfer of maternal immunity is beneficial to offspring for protection from infections. In our study, pups born to mothers during HI, showed increased resistance to infection with *Pcc* sporozoites, compared to pups born of naïve mothers. Antimalarial immunity can be transferred from the mothers to their young ones by antibodies in milk [61, 62], and antibody diversity can be influenced by antigen abundance and the duration of infection [63–65]. In our model, antigen exposure and infection duration are greatest in animals with HI, thus passive transfer of maternal antibodies might explain altered pup susceptibility in this group. More studies such as use of foster mothers and monitoring antibody levels in the pups will be needed to clearly demonstrate the mechanism of this protection.

To summarize, we have extended the *Pcc* model of pregnancy malaria in C57Bl/6 mice to incorporate recrudescences and HI, which are typical features of human PM. Mature *Pcc* parasites did not accumulate in the placental vasculature, a pattern more similar to *P*. *vivax* than *P*. *falciparum* in humans. *Pcc* infections in pregnant mice were associated with several poor outcomes, including maternal anemia, pregnancy loss, and persistent low weight and altered malaria susceptibility in offspring. HI with *Pcc* causes high levels of inflammatory cytokines, with the highest levels occurring in animals that experienced high parasite density and pregnancy loss. This model will be useful to interrogate the pathophysiological role of specific cytokines and immune mediators during PM that contribute to poor maternal and newborn outcomes.

## Supporting Information

S1 Fig Course of *P*. *chabaudi* infection in naïve non-pregnant C57BL/6 mice. (A) Course of peripheral parasitemia in mice infected intravenously with either *Pcc-*AS or *Pcc-*CB iRBCs. (B) Course of peripheral parasitemia in mice infected intravenously with 2000 sporozoites of either *Pcc-*AS or *Pcc-*CB strain. Data are presented as means with 95% confidence intervals.
(TIFF)
