# Supplementary figures and images for: Heterologous Infection of Pregnant Mice Induces Low Birth Weight and Modifies Offspring Susceptibility to Malaria
Source: PLoS One. 2016 Jul 28;11(7):e0160120. doi: 10.1371/journal.pone.0160120 (PMC4965193; doi:10.1371/journal.pone.0160120)

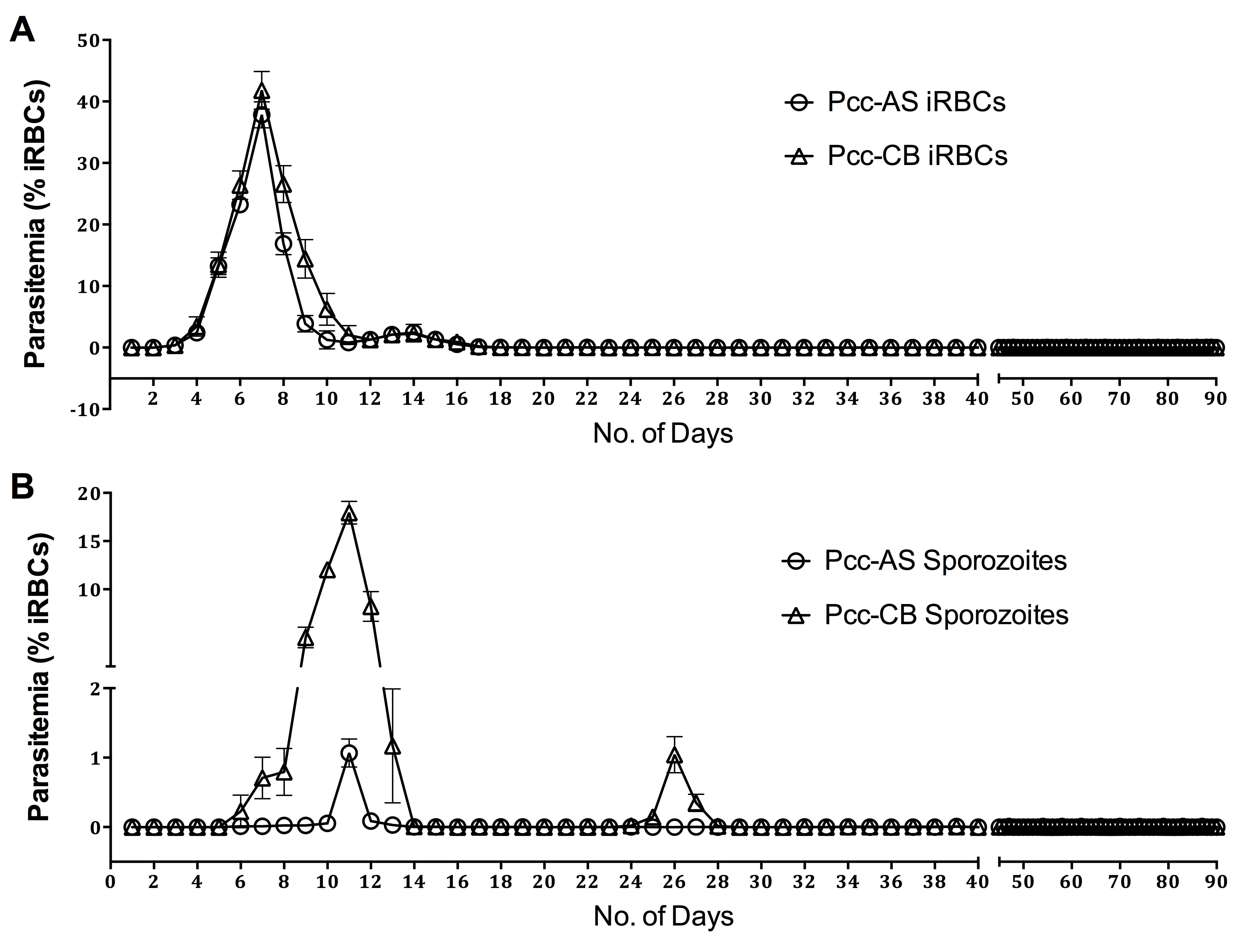

Supplement: S1 Fig — (A) Course of peripheral parasitemia in mice infected intravenously with either Pcc-AS or Pcc-CB iRBCs. (B) Course of peripheral parasitemia in mice infected intravenously with 2000 sporozoites of either Pcc-AS or Pcc-CB strain. Data are presented as means with 95% confidence intervals. (TIFF) [file pone.0160120.s001.tiff]
